# Compensation for impaired sensing of selenoprotein deficiency by alternative cysteine residues in KEAP1

**DOI:** 10.1016/j.redox.2026.104263

**Published:** 2026-06-15

**Authors:** Miu Sato, Takuya Iijima, Takafumi Suzuki, Masayuki Yamamoto

**Affiliations:** a Department of Biochemistry & Molecular Biology, Tohoku University Graduate School of Medicine, 2-1 Seiryo-machi, Sendai, Aoba-ku, 980-8573, Japan; b Department of Biochemistry & Molecular Biology, Tohoku Medical Megabank Organization, Tohoku University, 2-1 Seiryo-machi, Sendai, Aoba-ku, 980-8573, Japan; c Advanced Research Center for Innovations in Next-Generation Medicine (INGEM), Tohoku University, 2-1 Seiryo-machi, Sendai, Aoba-ku, 980-8573, Japan

**Keywords:** KEAP1, NRF2, Stress sensor, Selenoprotein deficiency, Electrophile

## Abstract

The KEAP1-NRF2 system is a master regulator of cellular defense against oxidative and electrophilic stresses. Cysteine residues within KEAP1 function as critical stress sensors. While KEAP1-Cys151 is a well-established sensor for electrophilic NRF2 activators, its contribution to the oxidative stress response remains unclear. Here, we investigated NRF2 activation in Cys151-deficient mice under hepatocyte-specific disruption of selenoprotein synthesis, a condition associated with profound redox imbalance. NRF2 activation and hepatic homeostasis were preserved in these mice, indicating that Cys151 is dispensable for sensing of selenoprotein deficiency. Conversely, loss of Cys226/Cys613-mediated sensing impaired NRF2 activation, leading to severe liver injury and lethality. Importantly, treatment with the Cys151-dependent electrophilic activator CDDO-Im restored NRF2 activity and improved survival in mice lacking functional Cys226/Cys613 sensing. Together, these findings demonstrate that individual KEAP1 cysteine residues have distinct functional roles in stress sensing, yet their signals converge on a common pathway to regulate NRF2 activation.

## 1 Introduction

Mammalian cells are continuously exposed to oxidative and electrophilic stresses, which impair cellular function by damaging nucleic acids, proteins, and membrane lipids. To overcome these stresses, cells are equipped with elaborate defense systems. The transcription factor NRF2 (nuclear factor erythroid 2-related factor 2) plays a central role in the inducible expression of cellular defense enzymes [1,2]. NRF2 dimerizes with small MAF proteins and binds to antioxidant/electrophile response elements (ARE/EpRE), now collectively referred to as CNC-sMAF binding elements (CsMBE), in the regulatory regions of cellular defense enzyme genes [[3], [4], [5]]. NRF2 target genes include those encoding detoxifying and antioxidant enzymes, metabolic enzymes, and heme and iron metabolizing enzymes [6]. Through these regulations, NRF2 protects cells against environmental and oxidative stresses and contributes to the maintenance of cellular homeostasis.

Under basal unstressed conditions, NRF2 is ubiquitinated by KEAP1 (Kelch-like ECH-associated protein 1)-CUL3 (Cullin 3) E3 ligase and rapidly degraded through the proteasomal pathway [[7], [8], [9]]. KEAP1 forms a homodimer and binds to a single NRF2 molecule [[10], [11], [12]]. KEAP1 interacts with Neh2 (NRF2-ECH homology domain 2) degron domain of NRF2 [7], and the two-site binding between the Neh2 domain and KEAP1 homodimer is critical for NRF2 ubiquitination by the KEAP1-CUL3 ubiquitin E3 ligase complex [10].

KEAP1 also acts as a cysteine-based sensor for electrophiles and oxidative stress. Upon exposure to electrophiles or oxidative stress, KEAP1 loses its ability to ubiquitinate NRF2, resulting in stabilization and nuclear accumulation of NRF2. KEAP1 is a cysteine-rich protein possessing 27 and 25 cysteine residues in the human and mouse proteins, respectively. Specific patterns of KEAP1 cysteine modifications by individual NRF2 activators have been identified [[13], [14], [15], [16], [17], [18], [19]]. Functional significance of the cysteine residues in KEAP1 has been examined by means of site-directed mutagenesis [[20], [21], [22], [23], [24], [25], [26], [27], [28], [29], [30]]. In particular, Cys226 and Cys613 have been identified as key sensors of hydrogen peroxide (H_2_O_2_) [25,31,32]. Cys226/613 residues were shown to be involved in formation of an intramolecular disulfide bond induced by H_2_O_2_ treatment [31,32].

Cys226/613 residues were also shown to be required for NRF2 activation in response to selenoprotein deficiency induced by conditional knockout of the *selenocysteine-tRNA* (*Trsp*) gene [28]. Deletion of the *Trsp* gene causes a failure of selenoprotein synthesis, leading to increased intracellular reactive oxygen species, including H_2_O_2_ [33,34], as well as enhanced protein disulfide bond formation, because the thioredoxin reductase-thioredoxin system serves as a major cellular disulfide-reducing pathway [35]. We have shown that the selenoprotein deficiency induces Cys226/613-mediated oxidation of KEAP1 and that the resulting redox imbalance can be alleviated by the induction of compensatory NRF2 activation [28,33]. Importantly, compound mutant mice lacking both *Trsp* and KEAP1-Cys226/Cys613 do not exhibit this compensatory NRF2 activation [28], indicating that Cys226/613 are required for sensing selenoprotein deficiency and triggering compensatory NRF2 activation.

Meanwhile, KEAP1-Cys151 has been identified as a key sensor for electrophilic NRF2 activators [[21], [22], [23], [24],^27^,^29^,^30^,^36^]. However, its role in oxidative stress sensing remains controversial. A study using a cell culture system revealed the intermolecular disulfide bond formation via Cys151 upon treatment with H_2_O_2_ [31], suggesting that this residue contributes to H_2_O_2_ sensing. In contrast, another study revealed that H_2_O_2_-induced NRF2 activation occurs even in cells expressing the KEAP1^C151S^ mutant [23], indicating that Cys151 is not essential for cellular H_2_O_2_ sensing. Thus, it is necessary to determine whether Cys151 plays a critical role in stress sensing *in vivo*. If Cys151 is specifically required for electrophile sensing, but dispensable for sensing signals arising from selenoprotein deficiency, it is conceivable that electrophiles could compensate for impaired Cys226/613-dependent NRF2 activation through intact Cys151. Such a compensatory mechanism, if present, could provide a potential strategy for the prevention or treatment of diseases associated with redox imbalance.

In this study, we examined the contribution of Cys151 to NRF2 activation in response to selenoprotein deficiency *in vivo.* Our results demonstrate that NRF2 activation and hepatic homeostasis are preserved in hepatocyte-specific selenoprotein-deficient mice lacking Cys151, indicating that Cys151 is dispensable for sensing selenoprotein deficiency. In contrast, loss of Cys226/613 impaired NRF2 activation in selenoprotein-deficient mice, resulting in severe liver injury and lethality. Importantly, administration of Cys151-dependent electrophilic activator CDDO-Im largely restored NRF2 activity and improved survival of the compound mutant mice. These findings indicate that KEAP1 utilizes distinct cysteine residues as independent sensors, Cys151 for electrophiles and Cys226/613 for selenoprotein deficiency. These sensors function in a complementary manner to respond to diverse environmental stresses, with their signals converging on NRF2 activation. Collectively, our findings support the concept that distinct KEAP1 sensors are differentially utilized and provide a mechanistic basis for the compensatory effects of electrophilic drugs under conditions of redox imbalance.

## 2 Methods

### 2.1 Mice

All mice were treated according to the regulation of The Standards for Human Care and Use of Laboratory Animals of Tohoku University (Sendai, Japan) and the Guidelines for Proper Conduct of Animal Experiments of the Ministry of Education, Culture, Sports, Science, and Technology of Japan. All animal experiments were executed with the approval of the Tohoku University Animal Care Committee. *Alb*Cre mice [37], *Trsp*^*fl*^ mice [33], *Keap1*^*C226S&C613S*^ mice [25], *Keap1*^*C151S*^ mice [28] have been described. The genotyping was performed by PCR or TaqMan real-time quantitative PCR (qPCR) (Life Technologies). Sequence information of the genotyping primers and probes are shown in Supplementary Table 1.

### 2.2 CDDO-Im administration

CDDO-Im (HY-15725; MedChemExpress) was first dissolved into dimethyl sulfoxide (DMSO), and the DMSO containing CDDO-Im was mixed with Cremophor-EL and phosphate buffered saline (PBS) at the ratio of 1:1:8 (DMSO: Cremophor-EL: PBS) and the final concentration of CDDO-Im in this mixture was 3 mmol/L [38]. The CDDO-Im mixture was administered at a dose of 10 mL per kg BW orally.

### 2.3 Western blot analysis

Livers of mice were homogenized in 9 vol of 0.25-M sucrose. For whole liver lysates, the homogenate was diluted with 2x sample buffer containing 20% glycerol, 4% SDS, 0.1-M Tris-HCl [pH 6.8], 12% 2-mercaptoethanol (2-ME), 0.88-mM phenylmethyl sulfonyl fluoride, cOmplete™ EDTA-free protease inhibitor cocktail tablet (Roche) and bromophenol blue. For nuclear fractionation, the homogenate was centrifuged at 600×g for 10 min. The pellet (nuclear fraction) was suspended in the 2x sample buffer. For redox Western blot, livers were homogenized in 4 vol of redox lysis buffer containing 0.1-M Tris-HCl [pH 8.0], 0.12-M NaCl, 0.2% deoxycholic acid, 5% IGEPAL® CA-630, 0.2-mM sodium fluoride, 0.2-mM EDTA, 0.1-mM phenylmethyl sulfonyl fluoride, 40-mM N-ethylmaleimide and cOmplete™ EDTA-free protease inhibitor cocktail tablet. The homogenate was diluted with 2 vol of 3x loading buffer containing 45% glycerol, 6% SDS, 0.2-M TrisHCl [pH 6.8] and bromophenol blue. Half of the sample was reduced by the addition of 2-ME (6% v/v). After heat denaturation, the protein samples were subjected to SDS-polyacrylamide gel electrophoresis (SDS-PAGE) and electro-transferred to PVDF membranes. Specific protein signals were detected by anti-NQO1 (ab2346, Abcam; 1:3000 dilution), anti-GPX1 [39]; 1:2000 dilution), anti-GPX4 (ab125066, Abcam; 1:1000 dilution), anti-α-TUBULIN (T9026, Sigma-Aldrich; 1:1000 dilution), anti-NRF2 ([40]; 1:1000 dilution), anti-LAMIN B1 (SC-374015, Santa Cruz; 1:1000 dilution), or anti-KEAP1 ([41]; 1:200 dilution) antibodies.

### 2.4 Gene expression analysis

Total RNA was extracted from livers of male mice using Sepasol®-RNA I Super G (09379-97; Nacalai Tesque). The RNA concentration was measured using a NanoPhotometer® NP80 (Implen). RNA was reverse transcribed into cDNAs using ReverTra Ace® qPCR RT Master Mix with gDNA Remover (FSQ-301; Toyobo) according to the manufacturer's instructions. The resulting cDNA was used as a template for reverse transcription qPCR (RT-qPCR) using a THUNDERBIRD® Probe qPCR Mix (QPS-101, Toyobo) with a QuantStudio (Life Technologies). The *Hprt* gene was used as an internal control. Gene-specific primer and probe sequences are shown in Supplementary Table 2.

### 2.5 Biochemical analysis

Plasma of the blood samples obtained from inferior vena cava were analyzed. Plasma alanine aminotransferase (ALT) values were determined using FUJI-DRI-CHEM 7000V (FUJIFILM).

### 2.6 Histological analysis

For hematoxylin-eosin (HE) staining, livers were fixed with Mildform® 10 N (131-10317; Wako Pure Chemical). The fixed tissues were embedded in paraffin, sliced into 4-μm-thick sections, and stained with HE.

### 2.7 Statistical analysis

Data were analyzed by one-way ANOVA followed by Tukey-Kramer HSD test using JMP® Student Edition 18.2.1 *P* < 0.05 was considered statistically significant; ∗*P* < 0.05, ∗∗*P* < 0.01, ∗∗∗*P* < 0.0001.

## 3 Results

**Fig. 1:**
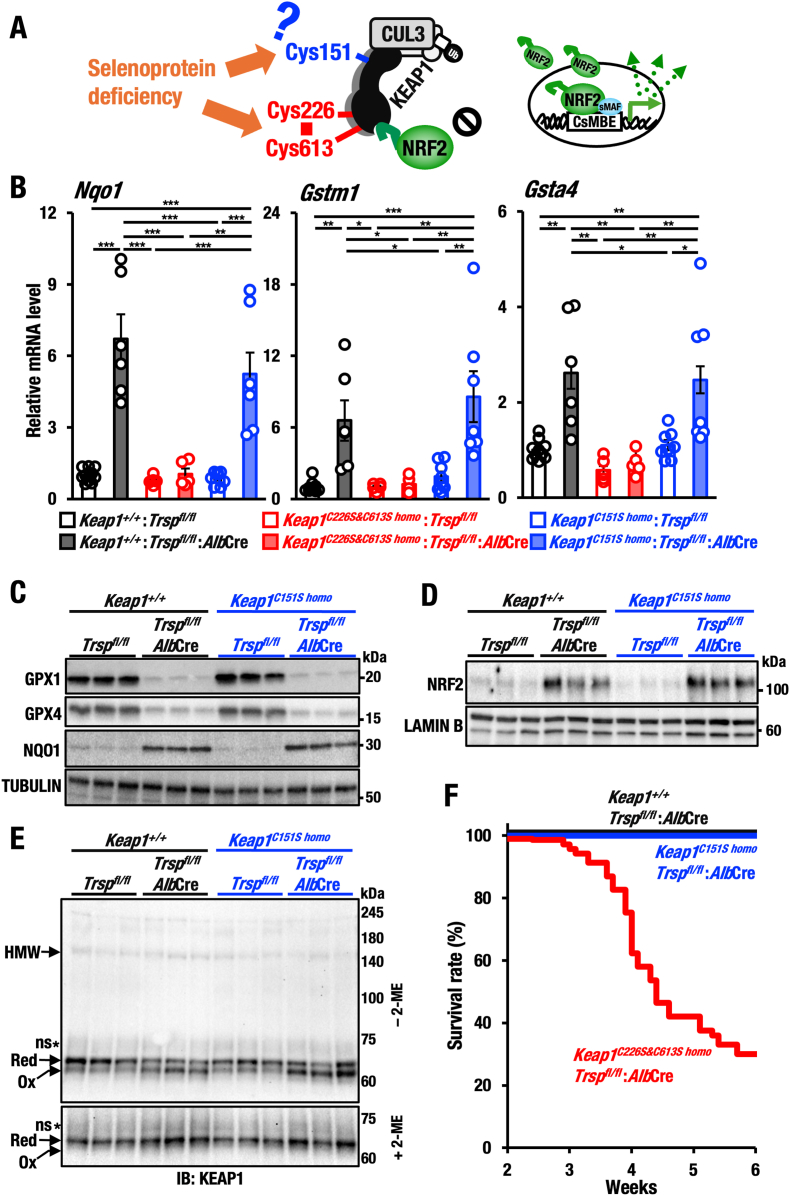
**KEAP1-Cys151 is dispensable for compensatory NRF2 activation in the selenoprotein deficient liver. (A)** Schematic illustration of the hypothesis that it remains unclear whether KEAP1-Cys151, an electrophile sensor, also plays a role in sensing selenoprotein deficiency. The disulfide bond shown between Cys226 and Cys613 represents a hypothetical model based on previous studies [25,31,32]. (**B**) Relative mRNA levels of *Nqo1*, *Gstm1* and *Gsta4* in livers of *Trsp*^*fl/fl*^ (n = 10), *Trsp*^*fl/fl*^:*Alb*Cre (n = 6), *Keap1*^*C226S&C613S homo*^:*Trsp*^*fl/fl*^ (n = 5), *Keap1*^*C226S&C613S homo*^:*Trsp*^*fl/fl*^:*Alb*Cre (n = 5), *Keap1*^*C151S homo*^:*Trsp*^*fl/fl*^ (n = 8) and *Keap1*^*C151S homo*^:*Trsp*^*fl/fl*^:*Alb*Cre (n = 7) mice were analyzed by RT-qPCR. Data were analyzed by one-way ANOVA followed by Tukey-Kramer HSD test (∗*P* < 0.05, ∗∗*P* < 0.01 and ∗∗∗*P* < 0.0001). (**C**) Protein levels of GPX1, GPX4 and NQO1 in whole liver lysates of *Trsp*^*fl/fl*^, *Trsp*^*fl/fl*^:*Alb*Cre, *Keap1*^*C151S homo*^:*Trsp*^*fl/fl*^ and *Keap1*^*C151S homo*^:*Trsp*^*fl/fl*^:*Alb*Cre mice at 2 weeks of age. (**D**) Nuclear NRF2 protein levels in livers of *Trsp*^*fl/fl*^, *Trsp*^*fl/fl*^:*Alb*Cre, *Keap1*^*C151S homo*^:*Trsp*^*fl/fl*^ and *Keap1*^*C151S homo*^:*Trsp*^*fl/fl*^:*Alb*Cre mice at 2 weeks of age. (**E**) Redox state of KEAP1 in livers of *Trsp*^*fl/fl*^, *Trsp*^*fl/fl*^:*Alb*Cre, *Keap1*^*C151S homo*^:*Trsp*^*fl/fl*^ and *Keap1*^*C151S homo*^:*Trsp*^*fl/fl*^:*Alb*Cre mice at 2 weeks of age were analyzed by Western blot with or without 2-mercaptoethanol (2-ME). HMW indicates high-molecular weight form of KEAP1 (approximately 150 kDa). Red and Ox represent reduced and oxidized forms of KEAP1, respectively. ns stands for a nonspecific band. (**F**) Survival of *Trsp*^*fl/fl*^:*Alb*Cre (n = 36), *Keap1*^*C226S&C613S homo*^:*Trsp*^*fl/fl*^:*Alb*Cre (n = 75) and *Keap1*^*C151S homo*^:*Trsp*^*fl/fl*^:*Alb*Cre (n = 51) cohorts of mice was monitored for 6 weeks after birth. The data were analyzed by the Kaplan-Meier method and compared using the log-rank test (*P* < 0.0001).

**KEAP1-Cys151 is dispensable for compensatory NRF2 activation in the selenoprotein deficient liver.** Cys226/613 and Cys151 of KEAP1 have been shown to play roles in sensing oxidative and electrophile stresses. Using *Trsp* knockout mice lacking selenoproteins, we demonstrated that Cys226/613 are indispensable for NRF2 activation in selenoprotein-deficient liver [28]. In contrast, while Cys151 is shown to be essential for sensing a group of electrophiles [22,23,36], it remains unclear whether the residue also plays roles in sensing selenoprotein deficiency (Fig. 1A).

In fact, it was reported that this Cys151 acts to sense H_2_O_2_ in an experiment using cultured cells [31]. Therefore, we wished to determine whether the Cys151 contributes to NRF2 activation in response to selenoprotein deficiency *in vivo*. For this purpose, in this study we generated compound mutant mice by crossing conditional hepatocyte-specific *Trsp* knockout (*Trsp*^*fl/fl*^:*Alb*Cre) mice with *Keap1*^*C151S*^ mutant mice. We hypothesized that if these compound mutant mice lacking both *Trsp* and KEAP1-Cys151 (*Keap1*^*C151S homo*^:*Trsp*^*fl/fl*^:*Alb*Cre) exhibit weaker level of NRF2 activation than that of *Trsp*^*fl/fl*^:*Alb*Cre mice, it will support the notion that the Cys151 also contributes substantially to sensing of selenoprotein deficiency caused by *Trsp* knockout (Fig. 1A).

To address this hypothesis, we first examined expression of canonical NRF2 target genes, including *NAD(P)H:quinone oxidoreductase 1* (*Nqo1)*, *Glutathione S-transferase mu 1* (*Gstm1*), and *Glutathione S-transferase alpha 4* (*Gsta4*). We found that mRNA levels of these genes were highly increased in the livers of *Trsp*^*fl/fl*^:*Alb*Cre mice in response to selenoprotein deficiency. In contrast, the increases were only marginal in the livers of compound mutant mice lacking both *Trsp* and KEAP1-Cys226/613 (*Keap1*^*C226S&C613S homo*^:*Trsp*^*fl/fl*^:*Alb*Cre) (Fig. 1B), indicating that Cys226/613 act as indispensable sensors of selenoprotein deficiency. In stark contrast, the expression levels of these NRF2 target genes were increased in the livers *of Keap1*^*C151S homo*^:*Trsp*^*fl/fl*^:*Alb*Cre mice to levels comparable to those observed in *Trsp*^*fl/fl*^:*Alb*Cre mice (Fig. 1B). The *Nqo1* mRNA level was slightly lower in the livers *of Keap1*^*C151S homo*^:*Trsp*^*fl/fl*^:*Alb*Cre mice than that in the livers of *Trsp*^*fl/fl*^:*Alb*Cre mice, but this was not statistically significant. These results thus do not support the hypothesis that the Cys151 sensor residue contributes to the NRF2 activation in response to selenoprotein deficiency in the liver.

We then examined the levels of selenoproteins, such as glutathione peroxidase 1 (GPX1) and glutathione peroxidase 4 (GPX4), and found that these protein levels were markedly lower in the livers of both *Trsp*^*fl/fl*^:*Alb*Cre and *Keap1*^*C151S homo*^:*Trsp*^*fl/fl*^:*Alb*Cre mice than those of control *Trsp*^*fl/fl*^ mice, indicating that the deletion of *Trsp* actually disrupts the expression of selenium-containing antioxidant enzymes. Importantly, the extent of reduction in these selenoproteins was comparable between *Trsp*^*fl/fl*^:*Alb*Cre and *Keap1*^*C151S homo*^:*Trsp*^*fl/fl*^:*Alb*Cre mice (Fig. 1C). The expression level of NQO1 protein, a representative NRF2 target gene product, was increased to comparable levels in the livers of *Trsp*^*fl/fl*^:*Alb*Cre and *Keap1*^*C151S homo*^:*Trsp*^*fl/fl*^:*Alb*Cre mice (Fig. 1C). In addition, NRF2 accumulated to a similar extent in the livers of both *Trsp*^*fl/fl*^:*Alb*Cre and *Keap1*^*C151S homo*^:*Trsp*^*fl/fl*^:*Alb*Cre mice (Fig. 1D). These results further support the conclusion that Cys226/613 are essential, but Cys151 is dispensable, for the compensatory NRF2 activation in selenoprotein-deficient liver.

**Cys151-dependent intermolecular disulfide bond formation does not contribute to the sensing of selenoprotein deficiency.** It has been reported that H_2_O_2_ treatment of cultured cells induces formation of intermolecular disulfide bonds through KEAP1-Cys151 [31]. To determine whether this is reproducible in selenoprotein-deficient livers *in vivo*, we examined redox state of KEAP1 in livers by redox Western blot analysis performed in the absence of the reducing agent 2-mercaptoethanole (2-ME). We detected no substantial increase in high-molecular-weight (HMW) bands corresponding to intermolecular disulfide bond formation in the livers of *Trsp*^*fl/fl*^:*Alb*Cre mice (Fig. 1E upper panel, HMW), indicating that the HMW bands are not induced by selenoprotein deficiency. Furthermore, the levels of these HMW bands were comparable in *Trsp*^*fl/fl*^:*Alb*Cre and *Keap1*^*C151S homo*^:*Trsp*^*fl/fl*^:*Alb*Cre mice, demonstrating that Cys151 is not involved in the formation of the HMW bands *in vivo*.

Meanwhile, as previous studies suggested [31,32], Cys226 and Cys613 may participate in redox-dependent sensing mechanisms and form an intramolecular disulfide bond under oxidative conditions. To address this hypothesis, we examined a fast-migrating band corresponding to oxidized KEAP1, which was previously shown to depend on Cys226/Cys613 in selenoprotein-deficient livers [28]. Consistent with our previous observations, the oxidized KEAP1 was significantly increased in the livers of *Trsp*^*fl/fl*^:*Alb*Cre mice. Importantly, we found that the oxidized KEAP1 was increased in the livers of *Keap1*^*C151S homo*^:*Trsp*^*fl/fl*^:*Alb*Cre mice to a similar level as that observed in *Trsp*^*fl/fl*^:*Alb*Cre mice (Fig. 1E upper panel, Ox), indicating that Cys151 residue is not involved in the formation of oxidized KEAP1. The oxidized form of KEAP1 disappeared upon treatment with a reducing agent (Fig. 1E lower panel), indicating that it contains a reversible disulfide bond, likely involving Cys226 and Cys613. These results indicate that reversible oxidation of Cys226/613, rather than Cys151, is associated with the sensing of selenoprotein deficiency.

**Cys151 sensor residue is dispensable for the survival of *Trsp* knockout mice.** One remaining possibility is that Cys151 contributes to sensing of selenoprotein deficiency in maintenance of homeostasis. To address this, we examined whether disruption of Cys151 affects mouse survival. To this end, we analyzed the survival of *Trsp*^*fl/fl*^:*Alb*Cre, *Keap1*^*C226S&C613S homo*^:*Trsp*^*fl/fl*^:*Alb*Cre, and *Keap1*^*C151S homo*^:*Trsp*^*fl/fl*^:*Alb*Cre mice. Kaplan-Meier analysis indicated that *Trsp*^*fl/fl*^:*Alb*Cre mice survived at least 6 weeks after birth (Fig. 1F, black line), while most *Keap1*^*C226S&C613S homo*^:*Trsp*^*fl/fl*^:*Alb*Cre mice died within 6 weeks after birth (red line). These results clearly indicate that the NRF2 activation compensates for selenoprotein deficiency, but this compensatory pathway is impaired in *Keap1*^*C226S&C613S homo*^:*Trsp*^*fl/fl*^:*Alb*Cre mice, consistent with our previous findings [28].

Of note, *Keap1*^*C151S homo*^:*Trsp*^*fl/fl*^:*Alb*Cre mice survived up to 6 weeks after birth (blue line), similar to *Trsp*^*fl/fl*^:*Alb*Cre mice. These results demonstrate that the Cys151 sensor residue is dispensable for the survival of *Trsp* knockout mice. Taken together, we conclude that selenoprotein deficiency is sensed by KEAP1 in a Cys226/613-dependent manner, but Cys151 does not contribute substantially to the sensing.

**Fig. 2:**
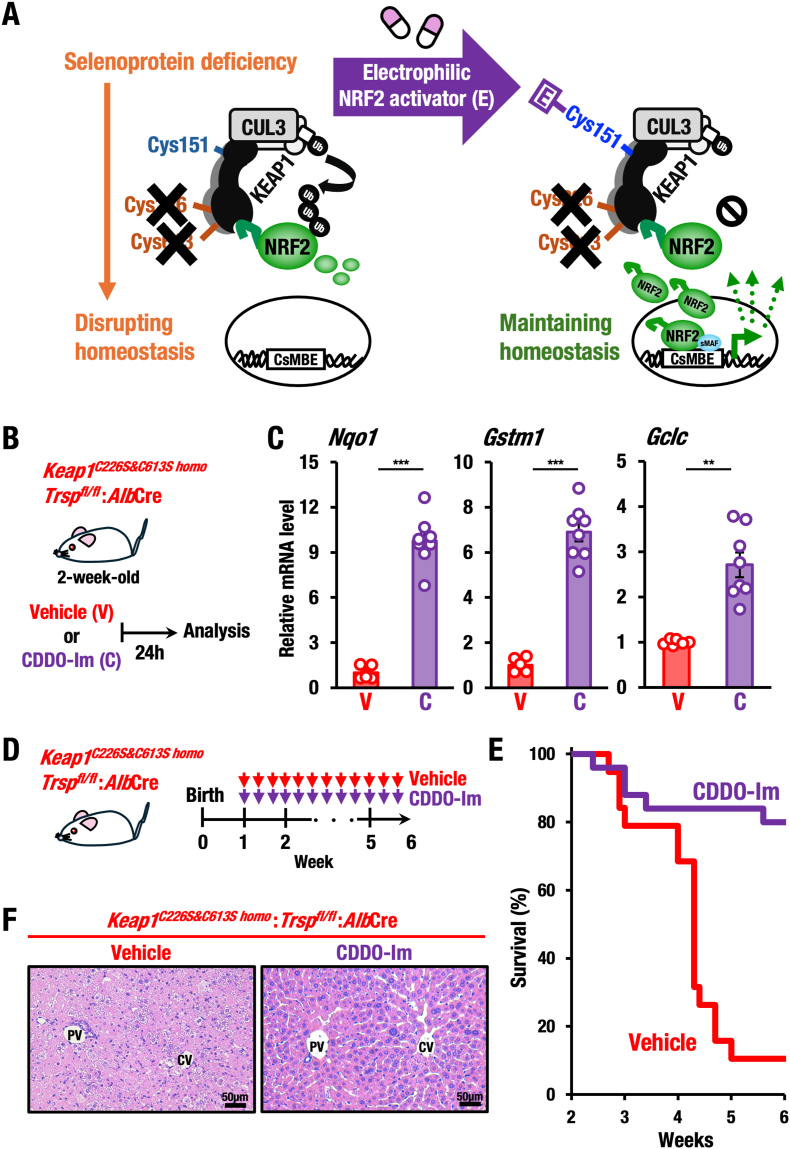
**CDDO-Im administration rescues the lethality of *Keap1*^*C226S&C613S homo*^:*Trsp*^*fl/fl*^:*Alb*Cre mice. (A)** Schematic illustration that pharmacological activation of NRF2 via intact KEAP1-Cys151 mitigates the effects arising from selenoprotein deficiency and restores hepatic homeostasis in *Keap1*^*C226S&C613S homo*^:*Trsp*^*fl/fl*^:*Alb*Cre mice. (**B**) *Keap1*^*C226S&C613S homo*^:*Trsp*^*fl/fl*^:*Alb*Cre mice were orally administered with vehicle (V) or CDDO-Im (C) (30 μmol/kg/body weight) at 2 weeks of age, and livers were collected 24 hours later. **(C)** Relative mRNA levels of *Nqo1, Gstm1* and *Gclc* in livers of vehicle- (n = 6) and CDDO-Im- (n = 8) treated *Keap1*^*C226S&C613S homo*^:*Trsp*^*fl/fl*^:*Alb*Cre mice were analyzed by RT-qPCR. The data were analyzed by two-tailed Welch's *t*-test (∗∗*P* < 0.01, ∗∗∗*P* < 0.0001). **(D)***Keap1*^*C226S&C613S homo*^:*Trsp*^*fl/fl*^:*Alb*Cre mice were orally administered vehicle or CDDO-Im (30 μmol/kg/body weight) three times per week from 1 to 6 weeks of age. **(E)** Survival of vehicle-treated (n = 19) and CDDO-Im-treated (n = 25) *Keap1*^*C226S&C613S homo*^:*Trsp*^*fl/fl*^:*Alb*Cre cohorts of mice was monitored for 6weeks after birth. The data were analyzed by the Kaplan-Meier method and compared using the log-rank test (*P* < 0.0001). **(F)** Representative images of hematoxylin and eosin (HE) staining of livers from vehicle- and CDDO-Im-treated *Keap1*^*C226S&C613S homo*^:*Trsp*^*fl/fl*^:*Alb*Cre mice. Pharmacological activation of NRF2 via KEAP1-Cys151 improved survival and attenuated hepatic injury.

**Electrophilic NRF2 activation via Cys151 pathway can compensate for impaired response to selenoprotein deficiency through Cys226/613 pathway.** *Keap1*^*C226S&C613S homo*^:*Trsp*^*fl/fl*^:*Alb*Cre mice failed to activate NRF2, resulting in markedly reduced survival due to disrupted hepatic homeostasis (Figs. 1F and 2A, left panel). Since KEAP1 uses Cys151 for sensing electrophilic NRF2 activators [22,23,36], we hypothesized that pharmacological activation of NRF2 through intact Cys151 sensor could alleviate the effects arising from selenoprotein deficiency and maintain hepatic homeostasis in the *Keap1*^*C226S&C613S homo*^:*Trsp*^*fl/fl*^:*Alb*Cre mice (Fig. 2A, right panel). To test this hypothesis, we orally administered CDDO-Im, a Cys151-dependent NRF2 activator, to the 2-week-old mutant mice and analyzed the mice 24 hours later (Fig. 2B). To our expectation, CDDO-Im treatment markedly induced the expression of *Nqo1*, *Gstm1*, and *Glutamate-cysteine ligase catalytic subunit* (*Gclc*) in the liver compared with the vehicle-treated mice (Fig. 2C), indicating that CDDO-Im induces NRF2 activation despite the loss of Cys226/613.

We next examined whether this NRF2 activation by CDDO-Im treatment or electrophilic activation of the NRF2 pathway could rescue the lethality of the *Keap1*^*C226S&C613S homo*^:*Trsp*^*fl/fl*^:*Alb*Cre mice, which was caused by selenoprotein deficiency. To address this question, we treated these mice with CDDO-Im three times per week from 1 to 6 weeks of age (Fig. 2D). Long-term CDDO-Im administration markedly improved survival of the *Keap1*^*C226S&C613S homo*^:*Trsp*^*fl/fl*^:*Alb*Cre mice compared with vehicle-treated mice (Fig. 2E). Approximately 80% of the mice treated with CDDO-Im survived 6 weeks, while untreated mutant mice died by 4 weeks of age. When we dissected a representative 3-week-old vehicle-treated mouse that euthanized due to waisted appearance, the mouse exhibited elevated plasma ALT level (16,700 U/L) and extensive hepatocyte death (Fig. 2F, left panel). Importantly, representative 6-week-old CDDO-Im-treated mice showed much lower ALT level (mean 54 U/L) and normal liver histology (Fig. 2F, right panel).

These results demonstrate that Cys151-mediated NRF2 activation is sufficient to compensate for impaired NRF2 activation in response to selenoprotein deficiency caused by Cys226/613 mutations, thereby protecting *Keap1*^*C226S&C613S homo*^:*Trsp*^*fl/fl*^:*Alb*Cre mice from severe tissue damage and juvenile lethality. The electrophilic NRF2 activation via Cys151 sensor fully sustains development of mice lacking Cys226/613-dependent sensing of selenoprotein deficiency. This finding supports the notion that distinct upstream stress-sensing mechanisms converge at the level of KEAP1 inactivation, whereas the downstream pathway leading to NRF2 activation is shared. These results highlight the therapeutic potential of activating NRF2 through alternative KEAP1 sensing mechanisms to compensate for impaired redox homeostasis and support the development of NRF2-targeted therapies for redox imbalance-associated diseases.

## 4 Discussion

**Fig. 3:**
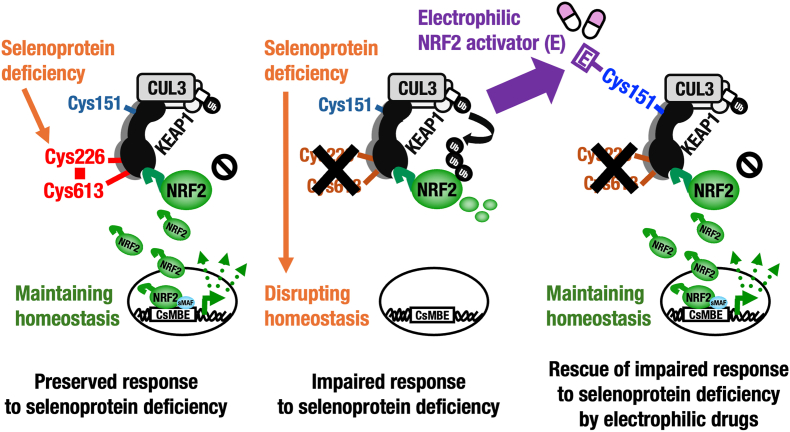
**KEAP1 cysteine sensor for electrophiles compensates for the impaired response to selenoprotein deficiency**. Under selenoprotein deficiency, Cys226/613 sense the stress and lead to NRF2 activation, resulting in the homeostasis maintenance (left panel). Under impaired response to selenoprotein deficiency due to unavailability of Cys226/613, KEAP1 fails to activate NRF2 and antioxidant response pathway, leading to disruption of homeostasis (middle panel). However, pharmacological activation of NRF2 through the intact Cys151 pathway effectively rescues the impaired response to selenoprotein deficiency (right panel).

The KEAP1-NRF2 system is a central regulator of cellular redox homeostasis, yet the functional annotations of individual KEAP1 cysteine sensor residues *in vivo* remain to be clarified. In this study, we demonstrate that Cys226/613, but not Cys151, are essential for compensatory NRF2 activation in response to selenoprotein deficiency in the liver. Conversely, as shown in Fig. 3, pharmacological activation of NRF2 through the intact Cys151 pathway effectively rescued the lethal phenotype associated with the disruption of Cys226/613-mediated NRF2 activation. These findings provide direct lines of evidence that KEAP1 employs distinct cysteine residues in response to diverse stresses, but the signals from the distinct sensor residues of KEAP1 converge at the level of KEAP1 inactivation or NRF2 activation.

Previous biochemical and cell-based studies suggested that multiple KEAP1 cysteine residues participate in the sensing of various stresses [23,25,42]. However, overlaps or mutual coverages of these multiple sensor pathways have not been examined extensively. Cys151 is widely recognized as one of the critical sensors for electrophilic NRF2 activators [22,23,36,43], whereas Cys226/Cys613 have been implicated in NRF2 activation in response to selenoprotein deficiency [28]. Our current *in vivo* analyses clearly show that Cys226/613 are indispensable for NRF2 activation in response to selenoprotein deficiency, whereas Cys151 is dispensable. Furthermore, we show here that pharmacological NRF2 activation via Cys151 using CDDO-Im can restore NRF2 target gene expression, improved liver integrity, and markedly extended survival of compound mutant mice lacking both*Trsp* and KEAP1-Cys226/613, indicating that electrophile-responsive sensors of KEAP1 can bypass defects in selenoprotein deficiency sensing pathways and restore adaptive NRF2 responses. Such cross-compensation highlights the modular nature of KEAP1-mediated regulation and suggests that different sensor cysteines converge on a common NRF2 stabilization mechanism. These results underscore the importance of evaluating KEAP1 sensor functions in physiologically relevant stress conditions, which will provide solid basis for the development of NRF2-activating drugs.

From a physiological perspective, selenoprotein deficiency represents a unique model of chronic stress characterized by impaired peroxide detoxification and disrupted redox buffering. The observation that Cys226/613, but not Cys151, govern NRF2 activation in this context supports the notion that distinct KEAP1 cysteine residues contribute differentially to the sensing of stress-related signals *in vivo*, rather than functioning as a uniform redox detection system.

It should be noted that electrophilic modification of Cys151 exerts a dominant inhibitory effect on NRF2 degradation. Recent co-crystal structure analyses of the KEAP1 BTB domain in complex with Cys151-dependent NRF2 activators revealed that ligand-induced subtle conformational changes within the BTB homodimer are key determinants of KEAP1-CUL3 E3 ligase activity [42]. Such ultra-sensitivity may explain the potent NRF2 activation elicited by electrophilic compounds even at low exposure levels. This mechanism appears to differ from that involved in Cys226/613-dependent NRF2 activation under conditions of selenoprotein deficiency [28]. In contrast, signaling pathways responding to chronic redox imbalance may need to accommodate a broader dynamic range of cellular perturbations.

In conclusion, our study provides *in vivo* evidence for the functional specialization of KEAP1 cysteine sensors and demonstrates that electrophile-responsive NRF2 activation can restore homeostasis when Cys226/613-dependent sensing pathways are compromised. These findings refine current models of KEAP1 regulation, emphasize the context-dependent nature of cysteine sensor utilization, and support the rationale for selectively targeting NRF2 activation pathways in redox-associated disease therapy and drug development.

## CRediT authorship contribution statement

**Miu Sato:** Conceptualization, Data curation, Formal analysis, Investigation, Validation, Visualization, Writing – original draft, Writing – review & editing. **Takuya Iijima:** Data curation, Investigation. **Takafumi Suzuki:** Conceptualization, Funding acquisition, Investigation, Project administration, Supervision, Writing – original draft, Writing – review & editing. **Masayuki Yamamoto:** Conceptualization, Funding acquisition, Investigation, Project administration, Supervision, Visualization, Writing – original draft, Writing – review & editing.

## Declaration of competing interest

The authors declare that they have no known competing financial interests or personal relationships that could have appeared to influence the work reported in this paper.

## Data Availability

No data was used for the research described in the article.
