# Supplementary material for: Compensation for impaired sensing of selenoprotein deficiency by alternative cysteine residues in KEAP1
Source: Redox Biol. 2026 Jun 15;95:104263. doi: 10.1016/j.redox.2026.104263 (PMC13311275; doi:10.1016/j.redox.2026.104263)
Supplement: Multimedia component 1 [file mmc1.docx]

**Supplementary Table 1**

| Oligo | Sequence | Experiment |
| --- | --- | --- |
| Cre1 | TGCCACGACCAAGTGACAGCAATG | PCR |
| Cre2 | AGAGACGGAAATCCATCGCTCG | PCR |
| SeCond F1 | GGCTGACTTACAGTTTCAGAGGCAC | PCR |
| SeCond R1 | ACCAACTCCCCTTGAGTTTAGACGC | PCR |
| C226S-F | CCAAGCAGGAGGAGTTCTTCAAC | TaqMan |
| C226S-R | GCAGCGTACGTTCAGATCATC | TaqMan |
| C226WT-P | VIC-TGTCACACTGCCAGCTG-MGB | TaqMan |
| C226S-P | FAM-CTGTCACACTCCCAGCTG-MGB | TaqMan |
| C613S-F | GTGAGGTGACCCGCATGAC | TaqMan |
| C613S-R | TTCAGCAGGTACAGTTTTGTTGATC | TaqMan |
| C613WT-P | VIC-ATGGAACCCTGTCGGAA-MGB | TaqMan |
| C613S-P | FAM-ATGGAACCCTCCCGGAA-MGB | TaqMan |
| C151S-F | AAGGCTTATTGAGTTCGCCTACA | TaqMan |
| C151S-R | CCACGCTGTCAATCTGGTACAT | TaqMan |
| C151WT-P | FAM-CAGGACACACTTCT-MGB | TaqMan |
| C151S-P | VIC-CAGGACCGACTTC-MGB | TaqMan |
