# Supplementary material for: Compensation for impaired sensing of selenoprotein deficiency by alternative cysteine residues in KEAP1
Source: Redox Biol. 2026 Jun 15;95:104263. doi: 10.1016/j.redox.2026.104263 (PMC13311275; doi:10.1016/j.redox.2026.104263)
Supplement: Multimedia component 2 [file mmc2.docx]

**Supplementary Table 2**

| Oligo | Sequence |
| --- | --- |
| mHprt-F | CTGGTGAAAAGGACCTCTCG |
| mHprt-R | TGAAGTACTCATTATAGTCAAGGG |
| mHprt-P | FAM-ATCCAACAAAGTCTGGCCTGTATCCAAC-TAMRA |
| mNqo1-F | AGCTGGAAGCTGCAGACCTG |
| mNqo1-R | CCTTTCAGAATGGCTGGCA |
| mNqo1-P | FAM-ATTTCAGTTCCCATTGCAGTGGTTTGGG-TAMRA |
| mGstm1-F | CCTATGATACTGGGATACTGGAACG |
| mGstm1-R | GGAGCGTCACCCATGGTG |
| mGstm1-P | FAM-CGCGGACTGACACACCCGATCC-TAMRA |
| mGsta4-F | GGGAACAGTATGAGAAGAAGATGCAAAA |
| mGsta4-R | CCCATCGATTTCAACCAAGG |
| mGsta4-P | FAM-TGCACACCTGCTTTTCGGCCAAG-TAMRA |
| mGclc-F | ATCTGCAAAGGCGGCAAC |
| mGclc-R | ACTCCTCTGCAGCTGGCTC |
| mGclc-P | FAM-ACGGGTGCAGCAAGGCCCA-TAMRA |
